# A novel missense mutation in complement factor I predisposes patients to atypical hemolytic uremic syndrome: a case report

**DOI:** 10.1186/s13256-022-03312-y

**Published:** 2022-03-04

**Authors:** Xin Wei, Juan Li, Xiaojiang Zhan, Luxia Tu, Haowen Huang, Ying Wang

**Affiliations:** 1 Nephrology Department, The First Affiliated Hospital of Nanchang University, Nanchang City, 330006 China; 2 Pathology Department, The First Affiliated Hospital of Nanchang University, Nanchang City, 330006 China; 3 Nanchang University, Nanchang City, 330031 China

**Keywords:** Atypical hemolytic uremic syndrome, Thrombotic microangiopathy, Alternative complement pathway, Complement factor I (CFI)

## Abstract

**Background:**

Atypical hemolytic uremic syndrome, also called the nondiarrheal form of hemolytic uremic syndrome, is a rare disease characterized by the triad of thrombocytopenia, Coomb’s test-negative microangiopathic hemolytic anemia, and acute renal failure. Approximately 60% of cases of atypical hemolytic uremic syndrome are associated with deficiencies of the complement regulatory protein, including mutations in complement factor H, complement factor I, or the membrane co-factor protein.

**Case presentation:**

We report the case of a 26-year-old Asian man who presented with pulmonary infection, elevated blood pressure, microangiopathic hemolytic anemia, thrombocytopenia, and acute renal failure. Renal biopsy revealed diffuse capillary fibrin deposition, endothelial swelling, and arteriole narrowing like “onion skinning” consistent with thrombotic microangiopathy. Bidirectional sequencing of CFH, CFHR5, CFHR1, CFI, DGKE, CFB, and MCP confirmed that the patient was heterozygous for a novel missense mutation, p.Cys67Phe, in CFI. This patient had rapid evolution to end-stage renal disease and needed renal replacement therapy. Plasma exchange seemed inefficacious in this patient.

**Conclusions:**

This report confirms the importance of screening patients with atypical hemolytic uremic syndrome for mutations in genes involved in complement system to clarify the diagnosis and demonstrates the challenges in the management of these patients.

## Background

Nondiarrheal or non-Shiga toxin-associated hemolytic uremic syndrome (HUS), also known as atypical HUS (aHUS), is a rare disease with incidence of 2 cases per million in adults, being predominantly related to complement regulatory protein deficiency [1]. aHUS commonly occurred in children and adolescents sporadically with a familial or relapsing pattern. A triggering event such as infection or pregnancy in a susceptible person leads to inappropriate activation of complement system, then causing aHUS [2]. Patients frequently progress to end-stage renal failure, in addition to many other extrarenal symptoms, and/or death. Nearly 70% of aHUS patients had mutations in genes coding for regulatory protein of complement system such as complement factor H (CFH), membrane cofactor protein (MCP)/CD46, CFI, CFB, complement component 3 (C3), complement factor H related 5 (CFHR5), thrombomodulin (THBD), and diacylglycerol kinase-epsilon (DGKE) [3]. Recently, additional genes have been implicated, such as the gene encoding plasminogen (PLG) [4], and more are likely to be discovered in the near future. Genetic testing for these mutations can be helpful for confirming the diagnosis of aHUS and predicting the prognosis.

It is reported that CFI mutations account for 4–10% of aHUS patients [5]. CFI is a serine proteinase that cleaves the α-chain of C3b and plays a key role in inhibition of the alternative pathway amplification loop that generates C3 convertase from C3b. Until now, 23 mutations in CFI have been reported in patients with aHUS, and their functional consequences have been characterized in approximately half of cases [6]. We report herein a novel missense mutation in the CFI that predisposed to aHUS in a sporadic patient.

## Case presentation

A young, 26-year-old Asian man presented to our hospital with acute renal failure and pulmonary infection on 25 November 2018. Approximately 10 days prior to presentation at our hospital, he began to show cough, expectoration, and stuffy chest. In the local hospital, they adopted cephalosporin antibiotics and etimicin (dose unknown) as anti-infective therapy. However, the above symptoms were not relieved, gradually developing into chest distress, shortness of breath, and oliguria. His first admission to our hospital was in the cardiovascular department. His blood pressure was 210/120 mmHg. Physical examination showed thick breathing sound and damp rales scattered in both lungs. Heart boundary was not big. This patient didn't show typical symptoms of peritonitis. Abdominal tenderness may be caused by ascites. Slight sunken edema of both lower limbs could be seen. Further physical examination did not show any significant findings, including skin and musculosketal system. The laboratory findings were as the follows: Tbil 32.7 µmol/L, iDBil 25.6 µmol/L, Scr 1524.5 µmol/L, Bun 39.5 mmol/L, UA 940 µmol/L, CK 686 U/L, lactate dehydrogenase (LDH) 2206 U/L, potassium 2.14 mmol/L, sodium 122.8 mmol/L, chlorine 79 mmol/L, calcium 1.75 mmol/L; IgG 5.96 g/L, IgA 1.91 g/L, IgM 0.34 g/L, C3 0.83 g/L, C4 0.29 g/L; white blood cell count 11.25 × 10^9^/L, hemoglobin 85 g/L, platelet 109 × 10^9^/L, neutrophil% 90.6%; PCT 4.03 ng/mL, ESR 140 mm/h, CRP 184 mg/L, activity of ADAMTS13 58.6%; proteinuria (+), urine RBC 6-9/Hp. Immunological and autoantibodies tests including rheumatoid factor, anti-cyclic citrullinated peptide, anti-double-stranded DNA antibody, anti-nuclear antibody, anti-neutrophil cytoplasmic antibody, anti-glomerular basement membrane antibody, anti-cardiolipin antibody, Ham’s test, and Coomb’s test were negative. Doppler ultrasound showed both kidneys of normal size (left 10.1 × 5.1 × 4.6 cm^3^, right 11.3 × 5.1 × 5.0 cm^3^), with cortex thickness of about 1.0 cm and decreased blood flow in both kidneys. Cardiac ultrasound showed larger left atrium with little effusion of pericardium. Chest computed tomography (CT) demonstrated that both lungs had multiple patchy shadows, indicating pneumonia. Ocular fundus examination showed optic papillary edema, hemorrhage, and exudation. No family history of the same disease was observed. Normal renal function and blood pressure were recorded in his most recent health examination report in August 2018. The primary diagnosis was acute renal failure, malignant hypertension, and pulmonary infection. The patient was transferred to the nephrology department for further treatment.

**Fig. 1:**
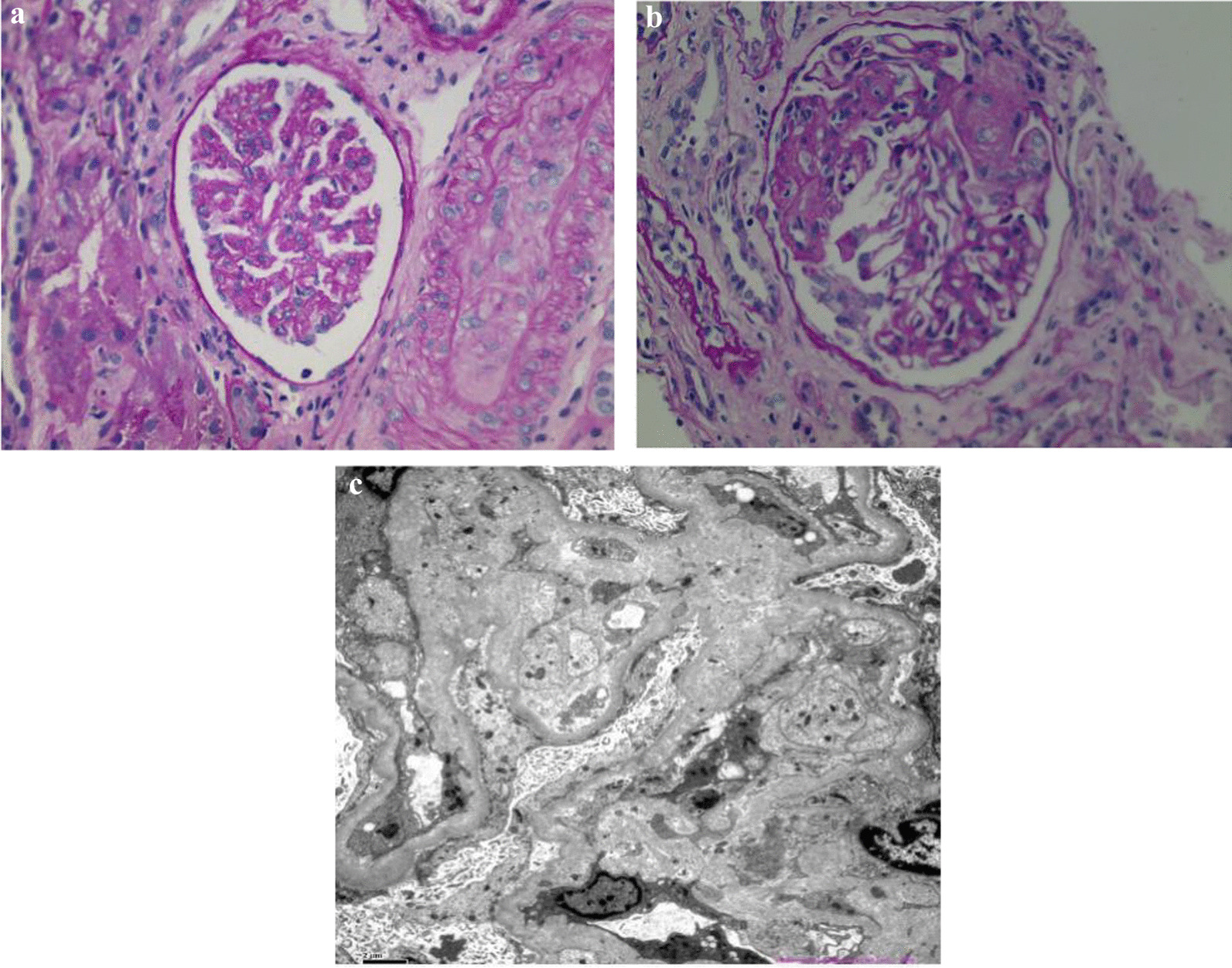
HE-stained biopsy (400×) reveals (**A**): glomeruli ischemia and shrinkage (left), and thickened wall of arterioles with occlusion lumen and “onion skin” appearance. **B** Another glomeruli shows endothelial swelling, capillary loop occlusion, and mild mesangial hyperplasia. Electron microscopy (**C**) shows expansion of subendothelial space with electrolucent material

After hemodialysis (CVVHDF, HDF, and HD, interspersed with average frequency of three times a week), antihypertension (nifedipine controlled-release tablets, terazosin hydrochloride, metoprolol, and irbesartan) and anti-infection treatment (cefoperazone–sulbactam), the patient’s symptoms such as chest distress and shortness breath improved, and the blood pressure decreased to 150–140/90–80 mmHg, but renal function did not recover. To clarify the cause of acute renal failure, renal biopsy was done ~ 10 days after admission to our hospital. The renal biopsy showed glomeruli ischemia and shrinkage, endothelial swelling, capillary loop occlusion and mild mesangial hyperplasia, renal interstitial edema, and multiple focal lymphmonocyte infiltration. The walls of arterioles and interlobular arteries were thickened with narrow lumen and “onion skin” appearance. Immunofluorescence microscopy displayed C3 (+), IgG (−), IgA (+/−), IgM (+) ,C1q (−), FRA (−), κ (−), λ (−). Electron microscopy revealed expansion of subendothelial space by electrolucent material. The pathological changes were consistent with thrombotic microangiopathies (Fig. 1).

**Fig. 2:**
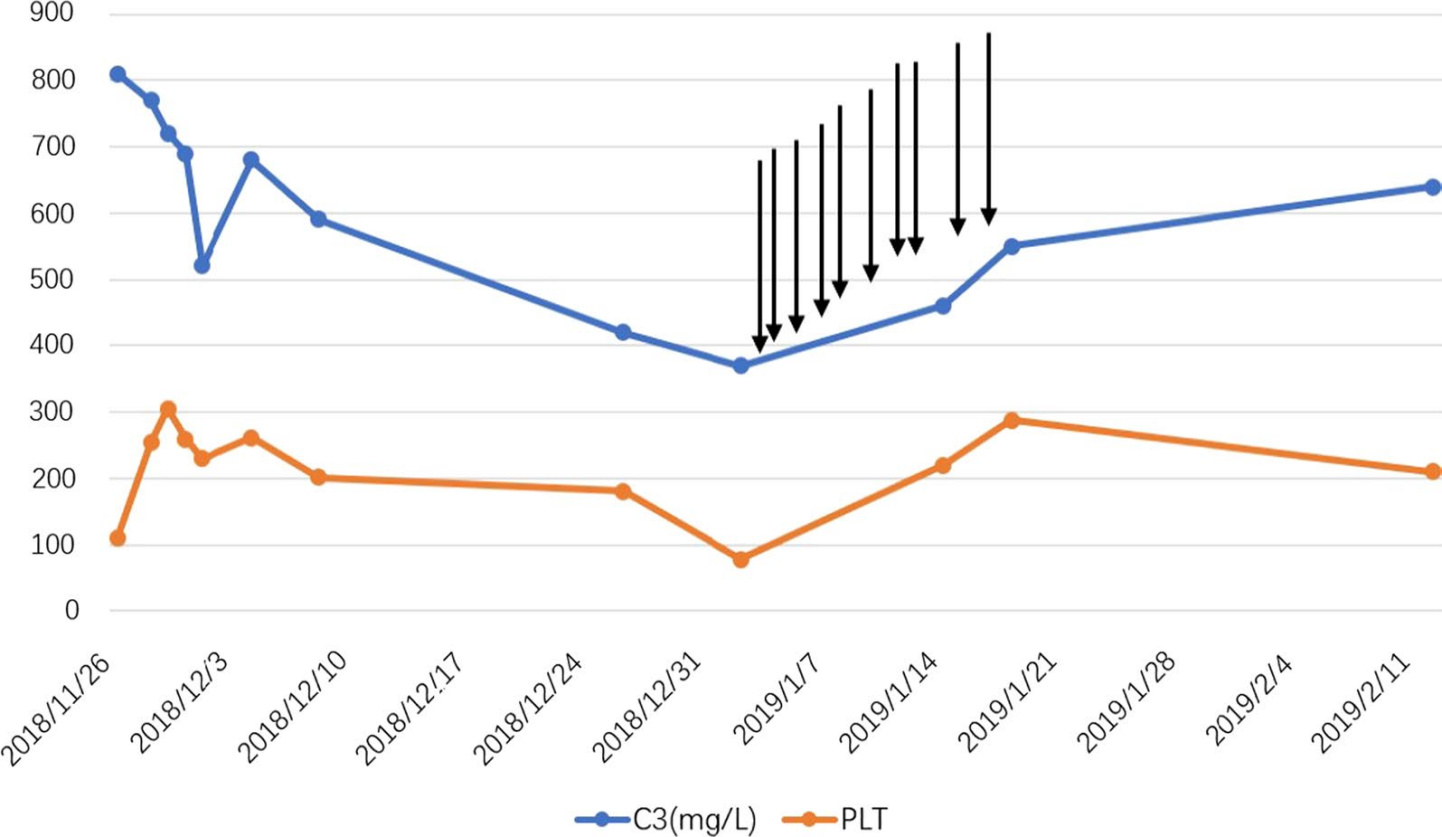
Platelet count and C3 level. Arrows indicate TPE treatments

While waiting for the renal pathological diagnosis, the serum level of C3, hemoglobin, and platelet count decreased rapidly, combined with elevated reticulocyte percentage and serum LDH level (Table 1). Despite lacking an excessive ratio of peripheral fragmented red cells, considering the low sensitivity of fragmented red cells, we make the presumptive diagnosis of complement-mediated hemolytic uremic syndrome. As eculizumab treatment could not be obtained, daily therapeutic plasma exchange (TPE) with thawed plasma was performed. The platelet count reached 220 × 10^9^/L after ten consecutive TPE treatments, accompanied by an increase of C3 and hemoglobin (Fig. 2).

**Table 1:** Patient’s laboratory values during hospital admission

	1st^a^	31th^a^	38th^a^	50th^a^	56th^a^	82th^a^
ALT (7–40 U/L)	16	14	11	8	6	7
AST (13–35 U/L)	33	6	10	14	15	14
Alb (40–55 g/L)	28.8	34.6	33.2	39.4	–	38.2
Tbil (3.42–20.5 µmol/L)	32	7.4	10.1	7.8	9.2	–
iDBil (0–6.84 µmol/L)	24.9	0.21	7.2	5.6	8.1	–
Scr (41–81 µmol/L)	1524.5	1280.4	914.2	860.3	735.5	732.7
Bun (3.1–8.8 mmol/L)	39.5	62.52	26.5	6.9	4.9	12.2
UA (155–357 µmol/L)	940	603	363	327	311	395
LDH (120–250 U/L)	2206	266	217	279	414	260
K^+^ (3.5–5.3 mmol/L)	2.14	6.3	5.56	4.04	3.53	4.38
Ca (2.11–2.52 mmol/L)	1.75	2.4	2.05	2.09	2.34	2.37
Hb (115–150 g/L)	85	97	79	77	82	85
Plt (125–350 × 10^9^/L)	109	189	78	220	287	210
C3 (0.79–1.53 g/L)	–	0.42	0.37	0.46	0.55	0.64
Proteinuria (0–0.15 g/24 h)	–	–	0.82	–	0.61	–
Urine volume (mL/day)	0	600	700	600	600	600

^a^Days post admission

*ALT* alanine aminotransferase, *AST* aspartate transaminase, *Alb* albumin, *Tbil* total bilirubin, *iDBil* indirect bilirubin, *Scr* serum creatinine, *Bun* blood urea nitrogen, *UA* uric acid, *LDH* Lactic dehydrogenase, *K+* kalium, *Ca* calcium, *Hb* hemoglobin, *Plt* platelet, *C3* complement 3

**Fig. 3:**
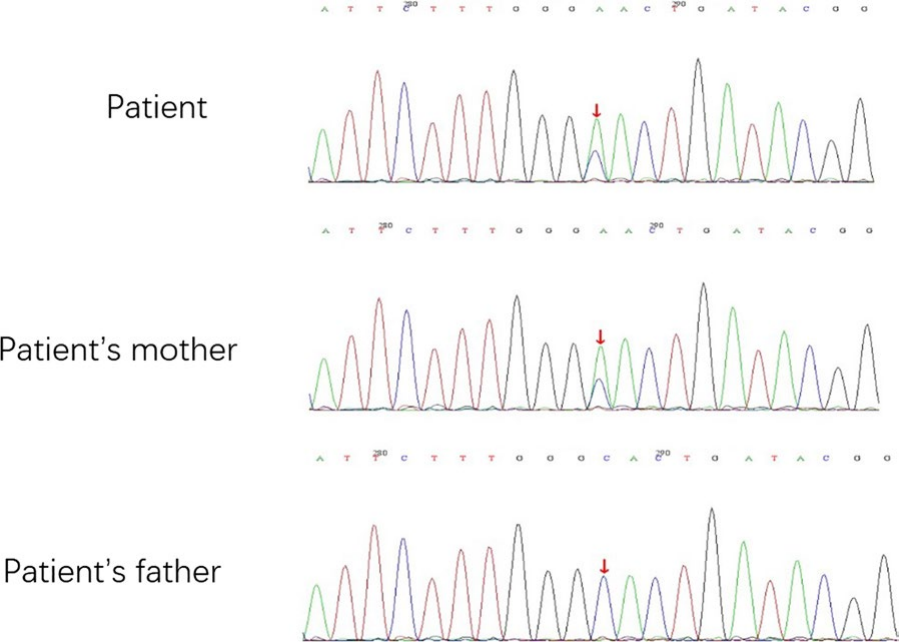
DNA sequencing data of the patient and his parent. In the patient and his mother, there is two peaks at the position of 200bp of CFI (red arrow) versus a single peak (red arrow) in his father's DNA sample, which indicates there is a missense mutant of G to A at the 200bp of CFI

Blood samples from this patient and his parents were sent for aHUS genetic panel sequencing, including CFH, CFHR5, CFHR1, CFI, DGKE, CFB, and MCP genes. Sequencing showed a novel heterozygous missense mutation in the CFI gene (NM_000204.2: c.200G>T), changing the 67th amino acid from cysteine to phenylalanine (p.C67F) (Fig. 3). Such mutation was also found in CFI of his mother but not his father’s CFI gene. At this point, the final diagnosis of this patient was aHUS. He was started on maintenance hemodialysis from then on. His platelet count and C3 level remained in normal range, while hemoglobin was about 100 g/L with treatment by erythropoiesis-stimulating agents.

## Discussion and conclusions

We report a patient who had pulmonary infection, malignant hypertension, acute renal failure with anemia, and thrombocytopenia, but normal C3 level, on first admission to our hospital. As the disease progressed, the C3 level and platelet count gradually decreased. Finally, renal biopsy and genetic sequencing helped to confirm the diagnosis of aHUS. Genetic sequencing showed a novel missense mutation in CFI, which was also found in his mother. However, she has not developed aHUS or any other complement-related diseases. After anti-infection and anti-hypertension treatment and intensive TPE, the patient’s infection and blood pressure were under control and the level of platelet count and C3 were normal, but his renal function did not recover, requiring hemodialysis as renal replacement therapy. We regularly followed up this patient, and discovered that his Scr level remained high under treatment of maintenance hemodialysis. This patient was very positive and cooperated with relevant treatment. However, his renal function developed to irreversible end-stage renal disease, with very poor prognosis. aHUS is an argent and life-threatening disorder, with up to 50% of patients progressing to ESRD and 25% dying during the acute phase. As nearly 70% patients can be identified with mutations of complement regulator proteins, the diagnosis of aHUS should be based on genetic sequencing and clinical findings [3]. Prior to mutation analysis, aHUS was diagnosed by excluding the following as causes: (1) thrombotic thrombocytopenic purpura (TTP), (2) Shiga-like toxin-producing *E. coli* (STEC-HUS), and (3) secondary thrombotic microangiopathies due to an underlying disease (e.g., malignant hypertension, drugs, and autoimmune disease) [7]. Knowing the affected gene can have prognostic value, so when aHUS is suspected, full screening for complement-associated genes (CFH, CFHR5, CFHR1, MCP, CFI, C3, CFB, THBD, DGKE, etc.) should be performed. Different affected complement components have different clinical course and outcome. Patients with CFH mutations generally have poor prognosis, with most patients progressing to ESRD or death within a year of presentation. In contrast, patient with MCP mutations are usually relapsing in nature. Patients with CFI mutations have a course that is intermediate between those with CFH and MCP mutations. Urgent plasma therapy and eculizumab is the main approach in these patients. The patient reported herein had normal C3 level and mild decreasing platelet count at first admission, which caused a delay in the diagnosis of aHUS, which in combination with the unavailability of eculizumab results in poor prognosis.

The gene encoding CFI is located on chromosome 4q25, which spans 63 kb and comprises 13 exons. The CFI protein is a serum serine protease, mainly involved in regulating the complement alternative pathway that mediates the cleavage of the α-chain of C3b leading to formation of iC3b, followed by its further degradation into C3dg and C3c. Furthermore, CFI can also cleave C4b to yield C4c and C4d [8, 9]. This leads to inhibition of the complement system and acts to protect host surfaces against complement activation. CFI mutations causing CFI deficiency were first described in 1970 and account for 4–10% of patients with aHUS [10]. Till now, nearly 23 mutations have been found in this gene with a predisposition to aHUS [11–14]. Eighty percent of mutations cluster in the serine protease domain [6]. Approximately 50% of mutants block protein secretion, while some mutants are secreted but have dysfunctional proteolytic activity with altered degradation of C3b/C4b in the fluid phase and on cell surfaces [15]. The different domains of CFI are well established: the second exon encodes a module only found in complement C6 and C7, the so-called membrane attack complex module [16]. The mutation we found in this patient is located in the second exon of CFI gene. Mutations in this exon may result in severe truncation of CFI and are probably not secreted. Such truncated CFI completely lacks the serine protease domain and cannot act as a protease [17].

The case report of this patient highlights the importance of gene sequencing for definitive diagnosis of genetic complement-mediated aHUS. A novel mutation in CFI was observed in this patient with aHUS. Further functional study should be conducted to understand the molecular mechanism corresponding to this mutation. Our experience with this case emphasizes rapid diagnosis and quick management, with targeted treatment with eculizumab as well as TPE being especially critical for recovery of renal function and/or survival of aHUS patients.

## Data Availability

The datasets used and analyzed during the current study are available from the corresponding author on reasonable request.

## Abbreviations

CFI: Complement factor I
aHUS: Atypical hemolytic uremic syndrome
CFH: Complement factor H
MCP: Membrane co-factor protein
CFHR1: Complement factor H related 1
CFHR5: Complement factor H related 5
CFB: Complement factor b
DGKE: Diacylglycerol kinase epsilon
ESRD: End-stage renal disease
THBD: Thrombomodulin
PLG: Gene encoding plasminogen
TTP: Thrombocytopenic purpura
TPE: Therapeutic plasma exchange
